# Application of the common base method to regression and analysis of covariance (ANCOVA) in qPCR experiments and subsequent relative expression calculation

**DOI:** 10.1186/s12859-020-03696-y

**Published:** 2020-09-29

**Authors:** Michael T. Ganger, Geoffrey D. Dietz, Patrick Headley, Sarah J. Ewing

**Affiliations:** 1 Department of Biology, Gannon University, Erie, PA 16541-0001 USA; 2 Department of Mathematics, Gannon University, Erie, PA 16541-0001 USA

**Keywords:** Confidence intervals, Linear relationship, Lognormal, qPCR analysis, Statistics

## Abstract

**Background:**

Quantitative polymerase chain reaction (qPCR) is the technique of choice for quantifying gene expression. While the technique itself is well established, approaches for the analysis of qPCR data continue to improve.

**Results:**

Here we expand on the common base method to develop procedures for testing linear relationships between gene expression and either a measured dependent variable, independent variable, or expression of another gene. We further develop functions relating variables to a relative expression value and develop calculations for determination of associated confidence intervals.

**Conclusions:**

Traditional qPCR analysis methods typically rely on paired designs. The common base method does not require such pairing of samples. It is therefore applicable to other designs within the general linear model such as linear regression and analysis of covariance. The methodology presented here is also simple enough to be performed using basic spreadsheet software.

## Background

The cells of an organism contain a large set of genes that encode information for constructing RNA and protein. Despite access to all of this information, individual cells may only transcribe a very small percentage of their genes [1]. Comparisons between unique cell types may show dramatic differences not only in the specific genes expressed but also in the expression level of commonly accessed genes [2]. Furthermore, expression levels are not expected to remain constant; in fact, our expectation is that expression levels will change in response to internal and external inputs, developmental state, and even disease state [3–5].

A central goal would be to elucidate a set of genes expressed and determine exactly how expression changes in response to external and internal signals and ultimately link this response to phenotypic changes. For this goal, quantification of gene expression could be performed in a variety of different ways via different methodologies [6], but the most common is to use differences in mRNA concentrations to quantify what is called relative expression that utilizes the polymerase chain reaction (PCR) to make detection of differences in initial RNA concentration possible [7]. Quantitative PCR (qPCR) has become the gold standard for such quantification and has become the technique of choice for diverse research questions [8–10].

The growth of amplicons within a qPCR reaction is expected to follow a logistic growth model where the increase in amplicons is exponential up until the point where reagents in the qPCR reaction begin to become limiting [8]. Because of this, Livak and Schmittgen [11] use the number 2 in their calculation of relative expression (equation 1) to indicate the potential for a doubling of the amplicon number each PCR cycle:

1 $$ Rel. Exp.={2}^{-\left[\left({C}_{q; GOI- Trea\mathrm{t} mentA}-{C}_{q; REF- Trea tmentA}\right)-\left({C}_{q; GOI- Trea tmentB}-{C}_{q; REF- Trea tmentB}\right)\right]}={2}^{-\left[\left(\Delta  {C}_{qA}\right)-\left(\Delta  {C}_{qB}\right)\right]}={2}^{-\Delta  \Delta  {C}_q} $$

This equation couples together the *C*_*q*_ values from Treatment A for both a gene of interest (GOI) and a reference gene (REF) and does the same for Treatment B. The difference in the exponent in *C*_*q*_ values for GOI and REF is referred to as a *∆C*_*q*_ value, and the difference between two *∆C*_*q*_ values as a *∆∆C*_*q*_ value [11].

From a theoretical perspective amplicons are expected to double each PCR cycle, yet many have shown that for various reasons this does not happen [12–14], and neglecting this fact can have measurable impacts on gene expression calculations [15, 16]. Others [15, 17] have developed methods for determining relative expression by incorporating a measure of the growth rate of a population of amplicons, called an efficiency value (E).

2 $$ Rel. Exp.=\frac{E_{GOI}^{-\left({C}_{q; GOI- TreatmentA}-{C}_{q; GOI- TreatmentB}\right)}}{E_{REF}^{-\left({C}_{q; REF- TreatmentA}-{C}_{q; REF- TreatmentB}\right)}} $$

Though not readily apparent in this formulation, the Pfaffl method equation (equation 2 [17]) also works with both *∆C*_*q*_ and ∆∆*C*_*q*_ values (see [15] for mathematical exposition).

**Fig. 1:**
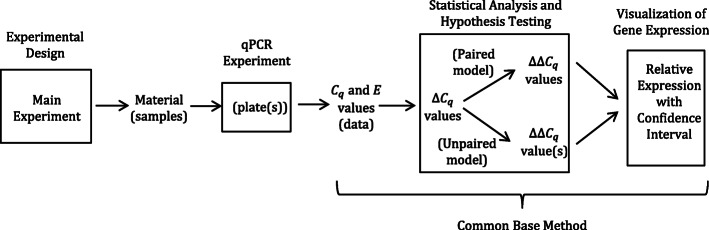
Work flow for an experiment showing main experiment, qPCR experiment, and the common base method (with statistical testing and the visualization of the relative expression value)

The technique of qPCR occupies a central position in the work flow, preceded by the design and execution of the main experiment and extraction of nucleic acid. qPCR is then followed by the analysis of data and finally the post-hoc calculation of a relative expression value (Fig. 1). Though these steps are separated by qPCR, they are in fact linked, in that experimental design dictates how gene expression should be analyzed and relative expression determined. It is worth noting that the commonly used models, specifically the ${2}^{-\Delta  \Delta  {C}_q}$ method [11] (2001; over 106,5000 citations as of March 2020) and the Pfaffl method [17] (2001; over 26,000 citations as of March 2020), were developed to analyze paired experimental designs. In this case, the experimental design is paired in nature, and so then would be the analysis. Paired models have their place and have proved very useful in determining expression of a gene 1) before and after treatment or 2) between two tissue types within the same organism. However, many types of experimental designs exist beyond paired designs that can be used to address a multitude of experimental questions. Such questions suggest the need for the development of alternative approaches.

**Fig. 2:**
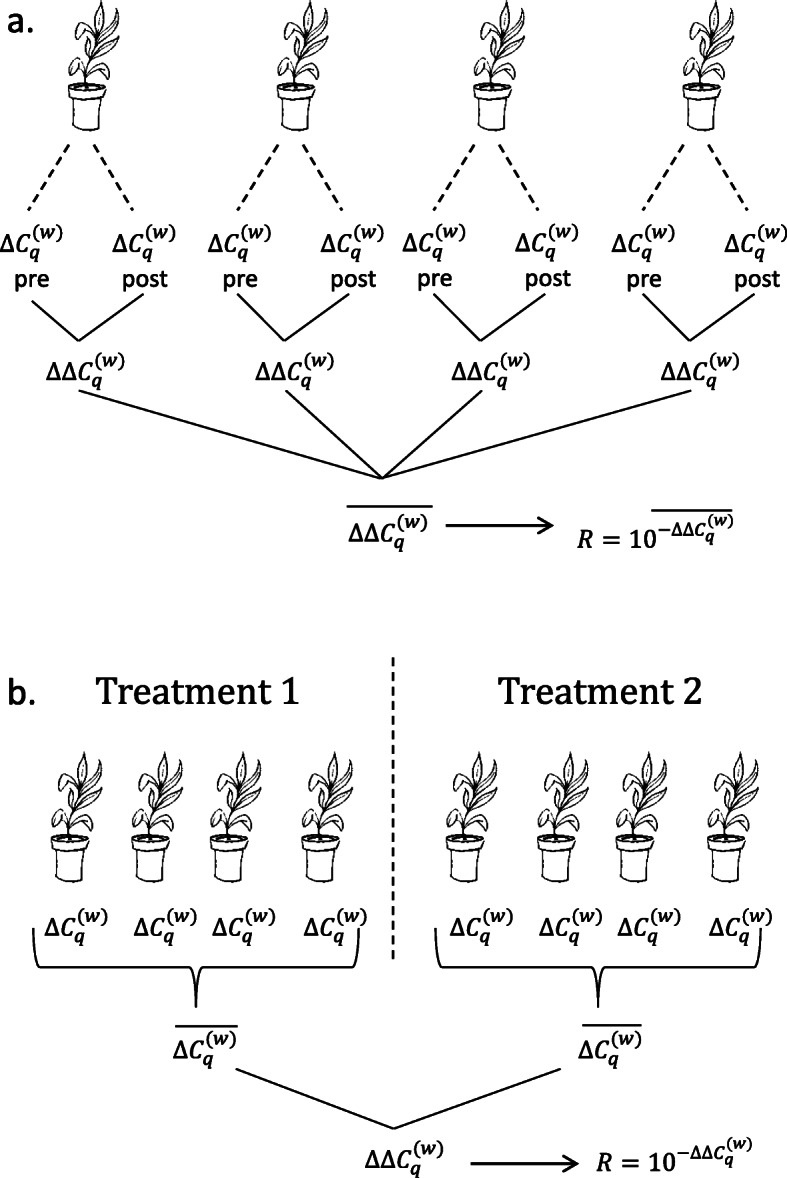
Representations of (**a**) paired model with associated $\Delta  {C}_q^{(w)}$ and $\Delta  \Delta  {C}_q^{(w)}$ values and (**b**) unpaired model with associated $\Delta  {C}_q^{(w)}$ and $\Delta  \Delta  {C}_q^{(w)}$ values where treatment variables are categorical

The common base method for the analysis of qPCR data [18] has inherent advantages over traditional methodologies and lends itself for use with other types of analyses within the general linear model (Fig. 2). Here we further develop statistical methodologies for unpaired models with a focus on linear relationships, specifically regression and analysis of covariance (ANCOVA). As with the common base method [18], we work with efficiency-weighted *∆C*_*q*_ values and develop relative expression calculations with associated confidence intervals post hoc.

### The Common Base method

The common base method calculations are kept in the logscale for as long as possible. Remaining in the logscale allows for the use of the more familiar arithmetic mean instead of the geometric mean and permits the use of parametric statistics [18]. Any choice of base for a logarithm may be made as long as it is used consistently. We have chosen to use base-10 logarithms throughout this work.

The common base method uses *C*_*q*_ and Efficiency (*E*) values to calculate an efficiency-weighted ${C}_q^{(w)}$ value. Let *r* denote a particular biological replicate, *t* denote a sample type, and *g* denote a particular gene (equation 3).

3 $$ {C}_{q;r,t,g}^{(w)}=\log \left({E}_{r,t,g}\right)\bullet {C}_{q;r,t,g} $$

The ${C}_{q;r,t,g}^{(w)}$ value is then normalized using a reference gene or genes, where *GOI* is the gene of interest and *REF* is a reference gene (equation 4 [18];).

4 $$ {\Delta C}_{q;r,t}^{(w)}={C}_{q;r,t; GOI}^{(w)}-\frac{1}{n}\sum \limits_{i=1}^n{C}_{q;r,t;{REF}_i}^{(w)} $$

The advantage of such values is that each efficiency-weighted *∆C*_*q*_ value can be treated separately in unpaired models that incorporate categorical and/or continuous variables. The major goal of our work here is to show that the common base method can be expanded to other statistical tools, including regression and analysis of covariance (ANCOVA). We will provide the mathematical approach for consideration of linear relationships, where at least one of the variables is ${\Delta  C}_q^{(w)}$, including calculation of $\Delta  \Delta  {C}_q^{(w)}$ values, relative expression ratios, and associated confidence intervals. We begin with regression and proceed into ANCOVA.

## Results

${\Delta  C}_q^{(w)}$ as the Dependent Variable.

We begin with consideration of the case where the dependent variable (*y)* is ${\Delta  C}_q^{(w)}$, while the independent is a non-gene expression variable (*x).* For example, consider the concentration of a hypothetical hormone α_1_ in plant leaves and expression of gene *G* in these same leaves, using ${\Delta  C}_q^{(w)}$ of *G*. We may be interested in how these two variables are related. For each individual, we could measure both α_1_ concentration and quantify, through qPCR, an efficiency-weighted *C*_*q*_ of gene *G* as ${\Delta  C}_q^{(w)}$. Suppose that all necessary assumptions for a regression (linearity, homoscedasticity, independence, and normality) have been met by our data set. Note that the assumptions of regression analysis are covered in any introductory statistics text.

Once the regression analysis has been performed, it is now possible to calculate relative expression ratios as a function of hormone concentration along with associated confidence intervals. As discussed earlier, in unpaired models $\Delta  {\Delta C}_q^{(w)}$ values are used to calculate relative expression ratios (*R*) after statistical analyses have occurred (Fig. 2).

**Fig. 3:**
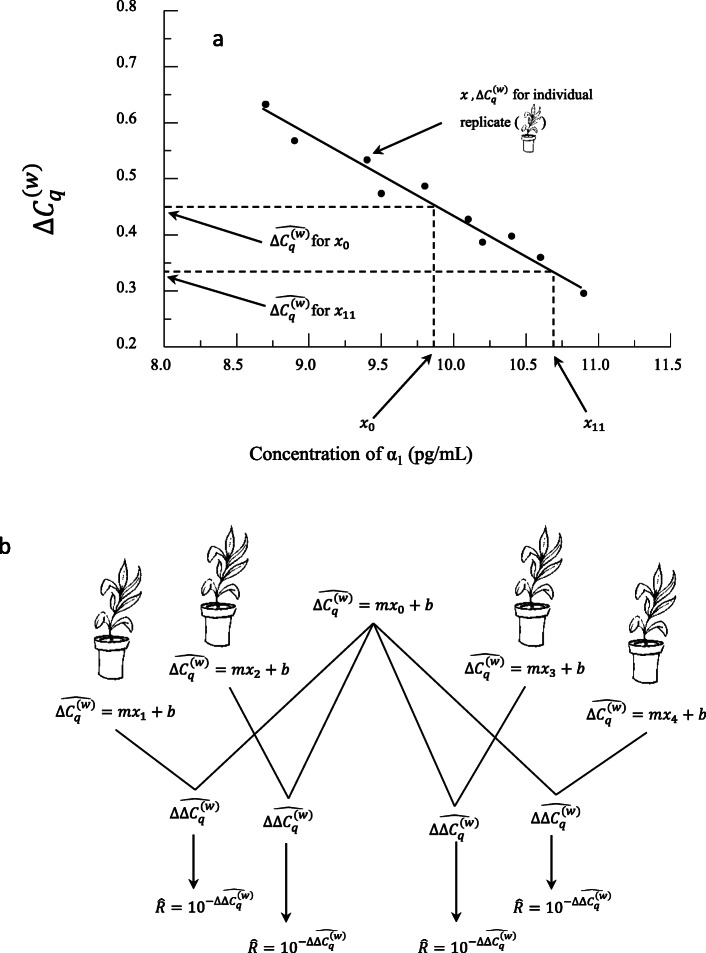
(**a**) Layout of regression showing variables used in calculations. (**b**) Representation of calculated $\hat{\Delta {\Delta C}_q^{(w)}}$and $\hat{R}$ values

Suppose the line of best fit is of the form.

5 $$ \hat{{\Delta C}_q^{(w)}}=\hat{y}= mx+b $$

where $\hat{y}$ is used to denote the predicted value of ${\Delta C}_q^{(w)}$ given a value of *x* based on the linear equation (Fig. 3a).

We can then rework the linear equation into a form that will yield an equation whose input is the concentration of hormone α_1_ and whose output is a relative expression ratio *R*. We first must choose a fixed input concentration of hormone α_1_ to be a “baseline” level (*x*_0_) for comparison. For our example, let *x*_0_ be the mean α_1_ concentration^1^ found in the original experiment. Let

6 $$ \hat{y_0}=m{x}_0+b $$

be the output predicted from the *x*_0_ concentration of hormone α_1_. We will now subtract (equation 6) from (equation 5) to produce an equation that outputs predictions for $\Delta  {\Delta C}_q^{(w)}$ values based on predicted ${\Delta  C}_q^{(w)}$ values and the choice of baseline *x*_0_ (Fig. 3b). In other words,

7 $$ \hat{\Delta {\Delta C}_q^{(w)}}=\left(\hat{{\Delta C}_q^{(w)}}\mathrm{for}\ x\right)-\left(\hat{{\Delta C}_q^{(w)}}\mathrm{for}\ {x}_0\right)=\hat{y}-\hat{y_0}=\left( mx+b\right)-\left(m{x}_0+b\right)=m\left(x-{x}_0\right) $$

where each $\Delta  {\Delta C}_q^{(w)}$ uses the baseline concentration of hormone α_1_ and varies the chosen concentrations of hormone α_1_ within the range of values used in the experiment (Fig. 3b). By applying an exponential function to (equation 7), we arrive at an exponential equation for relative expression ratio using the baseline. As a formula,

8 $$ \boxed{\hat{R}}={10}^{-\hat{\Delta {\Delta C}_q^{(w)}}}={10}^{-m\left(x-{x}_0\right)}\boxed{={10}^{m\left({x}_0-x\right)}} $$

**Fig. 4:**
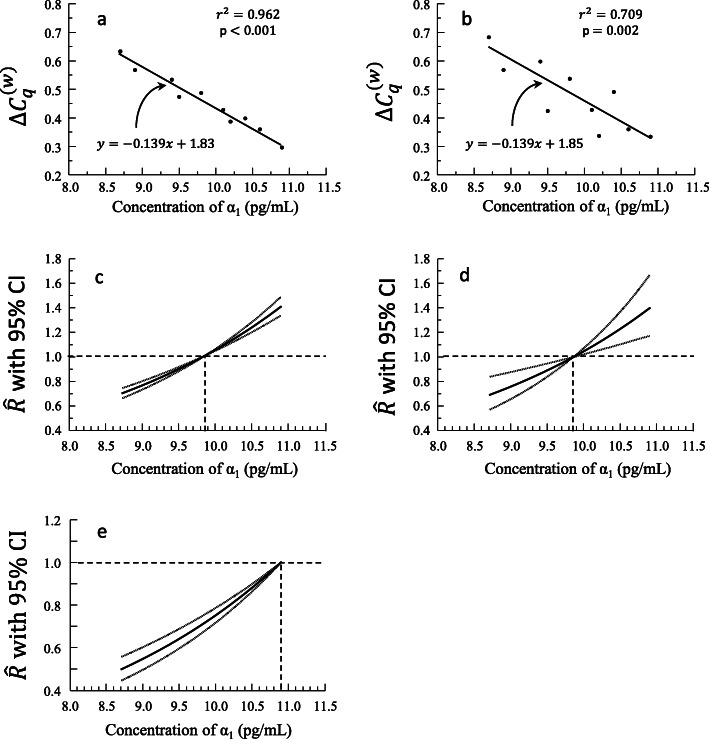
Results of regression analysis between concentration of hormone α_1_ and $\Delta  {C}_q^{(w)}$ where the two variables are (**a**) highly correlated (*r*^2^ = 0.962) and (**b**) correlated (*r*^2^ = 0.709). Plot of predicted relative expression ratios ($\hat{R}$) for (**c**) regression in A with 95% confidence interval (CI) and for (**d**) regression in B with 95% confidence interval (CI). (**e**). Plot of predicted relative expression ratios ($\hat{R}$) based on a linear regression between concentration of hormone α_1_ and ${\Delta C}_q^{(w)}$ with 95% confidence interval (CI). Relative expression and CI in (**c** and **d**) are based on comparisons to average concentration of hormone α_1_ measured, while (**e**) compares to the largest concentration of hormone α_1_ measured. Vertical dotted lines indicate *x*_0_

In other words, from a plot of ${\Delta C}_q^{(w)}$ and *x* (Figure 4a, Table 1), we have an equation that takes as input concentration *x* of hormone α_1_ and outputs a predicted $\hat{R}$ that is relative to the baseline concentration of α_1_ *x*_0_ (Figure 4c, Table 1). Notice that using *x* = *x*_0_ as the input in (equation 8) predicts a relative expression ratio of 1, which is exactly as it should be. We can predict that a plant with a hormone concentration of 8.85 pg/mL would have an expression of Gene *G* that is 27% ($\hat{R}=0.73\Big)$ lower than that of plants with average hormone concentration. (Any values for the independent variable may be chosen to predict *R* as long as they do not occur outside of the minimum and maximum values used in the study). It is important to note that relative expression plots tend to be inverse versions of ${\Delta  C}_q^{(w)}$ plots since high values of ${\Delta  C}_q^{(w)}$ indicate lower levels of gene expression than lower values.

**Table 1:** Hypothetical data used to generate Figure 4a where ${\Delta  C}_q^{(w)}$ is the dependent variable. Calculation of predicted relative expression, $\hat{R}$, values follows ${10}^{m\left({x}_0-x\right)}$, where *m* =  − 0.139, and these values are plotted in Figure 4c. *x*_0_ = 9.85 is the mean *x*. The 95% confidence interval for the slope *m* is (−0.162, −0.117)

α_1_ concentration (pg/mL)	${\Delta C}_q^{(w)}$		α_1_ concentration (pg/mL)	$\hat{R}$	Lower Confidence Interval	Upper Confidence Interval
8.7	0.633		8.7	0.692	0.651	0.734
8.9	0.568		8.9	0.738	0.702	0.774
9.4	0.534		9.1	0.787	0.756	0.817
9.5	0.474		9.3	0.839	0.815	0.862
9.8	0.487		9.5	0.894	0.878	0.910
10.1	0.428		9.7	0.953	0.946	0.960
10.2	0.387		9.9	1.016	1.014	1.019
10.4	0.398		10.1	1.083	1.070	1.098
10.6	0.360		10.3	1.155	1.129	1.183
10.9	0.296		10.5	1.231	1.191	1.274
			10.7	1.313	1.257	1.373
			10.9	1.399	1.327	1.479

### Confidence interval calculations from regression

While functions describing the relationship between two variables have great value, they only represent point estimates of output values for each input. However, assuming that the statistical assumptions for a valid regression have been met, one can also produce confidence intervals^2^ to envelope the point estimates resulting from the regression formula, allowing for meaningful error bars to be placed around point estimates. We will demonstrate that in order to calculate confidence intervals for relative expression value estimates, we first need to calculate the confidence intervals for $\Delta  {\Delta C}_q^{(w)}$. These confidence intervals are derived from the confidence interval around the regression slope *m*. Most statistical software tools (e.g., SPSS or Minitab), and even Excel, will compute the confidence interval for a regression slope as part of the standard regression output. This output is typically given as the low end and high end slope values of the 95% confidence interval in a form such as (*L,U*), though many tools allow for reporting of other confidence intervals. The formulas for *L* and *U* can be found in any introductory statistics textbook that covers inference related to linear regression.

We return to the setting where the concentration of hormone α_1_ *x* and ${\Delta C}_q^{(w)}$ value *y* are linearly related and fit a linear formula as in (equation 5). Let *x* be an arbitrary input value in the range of data values collected in your study, and let *x*_0_ be the fixed baseline input value with associated linear output as in (equation 6). In our example, we fix *x*_0_ to be the mean value of *x*, but any fixed choice will work. Recall from (equation 7) that $\hat{\Delta {\Delta C}_q^{(w)}}=m\left(x-{x}_0\right)$. Thus, the only random element in the estimate of $\hat{\Delta {\Delta C}_q^{(w)}}$ is the slope *m*, and so the uncertainty of $\hat{\Delta {\Delta C}_q^{(w)}}$ is solely a function of the uncertainty around *m*.

Suppose that the confidence interval (CI) on the slope parameter *m* is (*L,U).* Then the confidence interval for $\hat{\Delta {\Delta C}_q^{(w)}}$ is given by.

9 $$ \boxed{\mathrm{CI}\ \mathrm{for}\hat{\ \Delta {\Delta C}_q^{(w)}}=\left(L\left(x-{x}_0\right),U\left(x-{x}_0\right)\right)\ \mathrm{or}\ \left(U\left(x-{x}_0\right),L\left(x-{x}_0\right)\right)} $$

depending upon whether (*x* − *x*_0_) is positive or negative for each *x*. In order to calculate the corresponding confidence interval for the predicted relative expression ratio $\hat{R}$, we apply the exponential transformation to the interval calculated in (equation 9) (Fig. 4c) and mimic our end formula in (equation 8).

10 $$ \boxed{\mathrm{CI}\ \mathrm{for}\ \hat{R}=\left({10}^{L\left({x}_0-x\right)},{10}^{U\left({x}_0-x\right)}\right)\ \mathrm{or}\ \left({10}^{U\left({x}_0-x\right)},{10}^{L\left({x}_0-x\right)}\right)} $$

Depending upon whether (*x*_0_ − *x*) is positive or negative. (Notice the change in order of *x* and *x*_0_ made to match the order given in (equation 8).) From our example, the 95% confidence interval around our estimate of *R* given a hormone concentration of 8.85 pg/mL is 0.69–0.76 indicating relative expression of 69–76% compared to that of individuals with average hormone α_1_ concentration.

For any regression, *r*^2^ is an indication of the overall quality of the equation of the best fit line. Lower *r*^2^ values tend to increase the size of the confidence intervals around predicted relative expression ratios because as the *r*^2^ value lowers, the margin of error around the predicted slope value increases (Fig. 4b, d; Table 2).

**Table 2:** Hypothetical data used to generate Figure 4b. Calculation of predicted relative expression, $\hat{R}$, values follows ${10}^{m\left({x}_0-x\right)}$, where *m* =  − 0.139, and the values are plotted in Figure 4d. *x*_0_ = 9.85 is the mean *x*. The 95% confidence interval for the slope *m* is (− 0.212, − 0.066)

α_1_ concentration (pg/mL)	${\Delta C}_q^{(w)}$		α_1_ concentration (pg/mL)	$\hat{R}$	Lower Confidence Interval	Upper Confidence Interval
8.7	0.683		8.7	0.692	0.572	0.840
8.9	0.568		8.9	0.738	0.630	0.866
9.4	0.598		9.1	0.787	0.695	0.892
9.5	0.424		9.3	0.839	0.766	0.920
9.8	0.537		9.5	0.894	0.844	0.948
10.1	0.428		9.7	0.953	0.930	0.977
10.2	0.337		9.9	1.016	1.008	1.025
10.4	0.491		10.1	1.083	1.039	1.129
10.6	0.360		10.3	1.155	1.071	1.244
10.9	0.334		10.5	1.231	1.104	1.371
			10.7	1.313	1.138	1.511
			10.9	1.399	1.173	1.666

A Comment on Choosing the Baseline Value for the Independent Variable.

Notice that the widths of our confidence intervals are functions of the distance between input *x* and the baseline value *x*_0_ (equation 10). The uncertainty that leads to the error for the estimates is solely due to uncertainty in the slope *m*, which means that the choice in baseline value *x*_0_ does not alter the uncertainty. However, the choice of *x*_0_ does play a role in how that uncertainty is translated into a confidence interval around a given $\hat{\Delta {\Delta C}_q^{(w)}}$. As such, choosing *x*_0_ to be the mean value for *x* will result in overall smaller error bars and more symmetrically distributed error bars around estimates compared to choosing *x*_0_ to be one of the extreme values (minimum or maximum) (Fig. 4e; Table 3).

**Table 3:** Calculation of predicted relative expression, $\hat{R}$, values using hypothetical data from Table 1. Calculation of $\hat{R}$ values follows ${10}^{m\left(x-{x}_0\right)}$, where *m* =  − 0.139, and these values are plotted in Figure 4e. *x*_0_ = 10.9 is the largest *x* value. The 95% confidence interval for the slope *m* is (− 0.162, − 0.117)

α_1_ concentration (pg/mL)	$\hat{R}$	Lower Confidence Interval	Upper Confidence Interval
8.7	0.495	0.440	0.553
8.9	0.527	0.474	0.583
9.1	0.562	0.511	0.616
9.3	0.599	0.551	0.650
9.5	0.639	0.593	0.686
9.7	0.681	0.639	0.724
9.9	0.726	0.689	0.764
10.1	0.774	0.742	0.806
10.3	0.825	0.799	0.851
10.5	0.880	0.861	0.898
10.7	0.938	0.928	0.948
10.9	1	1	1

The selection of *x*_0_ should always be influenced by the experimental design. In our example, we selected the mean value of *x* for the baseline value *x*_0_ since values of hormone *α*_1_ concentration and ${\Delta  C}_q^{(w)}$ values were determined from randomly chosen plants. Suppose, however, that there is a tendency for the variable *x* to take on a certain value *x*_0_ in nature. If your experiment is to test the effects on gene expression by varying or manipulating the value of *x,* then it may make better sense to use the unmanipulated value *x*_0_ as the baseline in your calculations instead of the mean value of *x*, as that value serves as a natural point of comparison in your experiment. Such decisions should be made prudently.

In the absence of any other motivating factors or when the values of the independent variable will not be manipulated in the course of the experiment, we generally advocate choosing the mean value of *x* as the baseline value *x*_0_.

### A comment on slope of the regression line

The *p*-value in a linear regression is used to test the null hypothesis *m* = 0. In our example above, we were able to reject the null hypothesis and obtained the formula (equation 8) as a result. Notice that if we were unable to reject the null hypothesis, we would be left with the assumption that the slope is not significantly different from zero, and (equation 6) would result in the constant function $\hat{y}=b$, meaning that we have no evidence that the concentration of *α*_1_ has any effect on gene expression. (Equation 8) would yield $\hat{R}=1$, showing that changes in *α*_1_ concentration have no impact on the relative expression ratio for the gene in question.

${\Delta  C}_q^{(w)}$ as the Independent Variable.

It may be of interest to determine the effect of the expression of a gene on some measureable quantity (*y*). Such an approach is common in experiments where the level of expression of a gene is explicitly manipulated either by varying the strength of the promoter or varying the number of gene copies. The result would be two values for each individual, the efficiency-weighted ${\Delta  C}_q^{(w)}$ for a particular gene or gene array and a response variable, *y*. For example, suppose that a particular gene’s expression is thought to correlate with promiscuity in a certain species of animal as measured by time (min.) spent huddling with their partner (conceptual example derived from [19]). In this case, we would be using ${\Delta  C}_q^{(w)}$ values as the independent variable *x*, and *y* (time spent huddling) would be the dependent. The mathematics for this case is the inverse of the case above.^3^

**Fig. 5:**
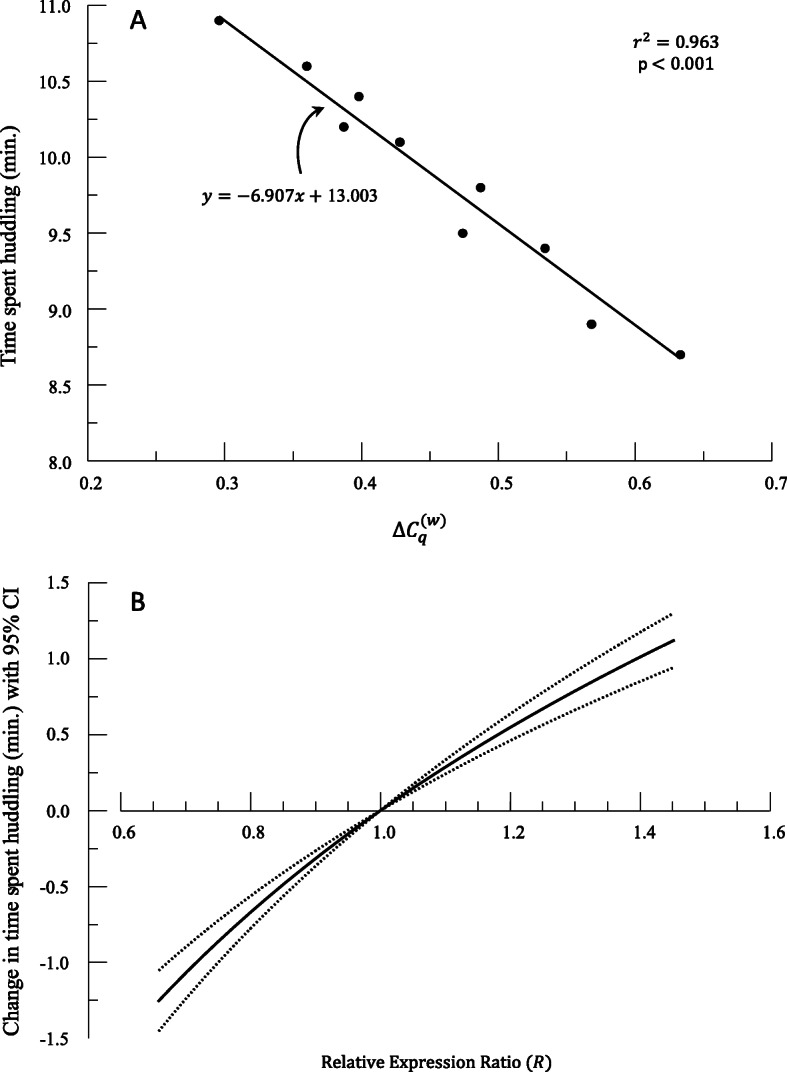
(A) Results of regression between $\Delta  {C}_q^{(w)}$ and T ime spent huddling. (B) Plot of predicted change in time spent huddling (min.) with 95% confidence interval (CI) based on relative expression ratio. *x*_0_ value is the average *x*

Suppose that the assumptions for a valid linear regression have been met and produce a line of best fit with associated statistics (Fig. 5a, Table 4).

11 $$ \hat{y}=m\ast {\Delta  C}_q^{(w)}+b= mx+b $$

**Table 4:** Hypothetical data used to generate Fig. 5a. Calculation of predicted huddling time, $\hat{y}$, values follows $\hat{y_0}-m\log (R)$, where *m* =  − 6.907, and these values are plotted in Fig. 5b. *x*_0_ = 0.457 is the mean *x*, and $\hat{y_0}=9.847$. The 95% confidence interval for the slope *m* is (−8.011, −5.803)

${\Delta C}_q^{(w)}$	Time spent huddling (min.)	${\Delta \Delta C}_q^{(w)}$	*R*	$\hat{y}$	Lower Confidence Interval	Upper Confidence Interval
0.633	8.7	0.177	0.666	8.628	8.433	8.823
0.568	8.9	0.112	0.774	9.077	8.954	9.200
0.534	9.4	0.0775	0.837	9.312	9.226	9.397
0.474	9.5	0.0175	0.961	9.726	9.707	9.745
0.487	9.8	0.0305	0.932	9.636	9.603	9.670
0.428	10.1	−0.0285	1.068	10.044	10.012	10.075
0.387	10.2	−0.0695	1.174	10.327	10.250	10.404
0.398	10.4	−0.0585	1.144	10.251	10.187	10.316
0.360	10.6	−0.0965	1.249	10.514	10.407	10.620
0.296	10.9	−0.1605	1.447	10.956	10.778	11.133

To calculate a functional form that involves relative expression ratios *R* and confidence intervals, one should judiciously choose a baseline value for gene expression ${\Delta  C}_q^{(w)}$, which we label as *x*_0_ for brevity. We set

12 $$ {\Delta \Delta  C}_q^{(w)}=x-{x}_0=\Delta  {C}_q^{(w)}-{x}_0 $$

and have $\hat{y_0}=m{x}_0+b$. As relative expression ratio $R={10}^{-\Delta {\Delta  C}_q^{(w)}}$, we can solve for ${\Delta \Delta  C}_q^{(w)}$ in terms of *R* to see that

13 $$ {\Delta \Delta  C}_q^{(w)}=-\log (R) $$

Therefore, subtracting $\hat{y_0}=m{x}_0+b$ from (equation 11) yields the formula

14 $$ \hat{y}-\hat{y_0}=m\left(x-{x}_0\right)=m{\Delta \Delta C}_q^{(w)}=-m\ast \log (R) $$

We can rearrange that into a final form by adding $\hat{y_0}$ to both sides of the equation

15 $$ \boxed{\hat{y}=\hat{y_0}-m\ast \log (R)} $$

(Equation 15) tells us that for a given *R*, or relative expression ratio between two values (*x* and *x*_0_), we expect a specific change in time spent huddling (Fig. 5b, Table 4). In our hypothetical case, individuals with 50% higher expression of the promiscuity gene (*R* = 1.5) have an increase in huddling time of 73.0 s. Note that this value is only applicable to a comparison with the currently chosen *x*_0_; in other words, a 50% increase in expression relative to *x*_0_. If you require a different set of comparisons, then you will require a new baseline for comparison.

As with all predictions of *y*, we recommend confidence interval calculations. We can generate formulas for confidence intervals to place around predicted values of the dependent variable given values of *R.* Suppose that the confidence interval on the slope parameter *m* is *(L, U).* Substitute this expression into (equation 15) and simplify to calculate a confidence interval for $\hat{y}$ based on a specified value of *R*.

16 $$ \boxed{\mathrm{CI}\ \mathrm{for}\ \hat{y}=\left(\hat{\ {y}_0}-U\ast \log (R),\hat{y_0}-L\ast \log (R)\ \right)} $$

where the order of *L* and *U* is swapped because of the negative multiplier in the formula. Given our hypothetical example above, the 95% CI for huddling time given a 50% increase in expression would be an increase in huddling time of 61.3 s – 84.6 s.

${\Delta  C}_q^{(w)}$as Both Independent and Dependent Variable.

Another useful technique might be to relate ${\Delta  C}_q^{(w)}$ values for two separate genes. This case is the intersection of the two cases listed above, but we include the derivation to make it explicit. The resulting regression would allow us to establish that the ${\Delta  C}_q^{(w)}$ of one gene is related to the ${\Delta  C}_q^{(w)}$ of a second gene. We may choose one of the gene’s ${\Delta  C}_q^{(w)}$ values to represent the independent variable (gene A) and the other’s ${\Delta  C}_q^{(w)}$ values to represent the dependent variable (gene B). The resulting model will show how a specific ${\Delta  C}_{q;A}^{(w)}$ value for gene A can be used to predict a ${\Delta  C}_{q;B}^{(w)}$ value for gene B. One can then also place a confidence interval around that prediction. On the other hand, one can swap the positions of the genes to make predictions of ${\Delta  C}_{q;A}^{(w)}$ values for gene A given ${\Delta  C}_{q;B}^{(w)}$ values for gene B and similarly place confidence intervals around the predictions. The choice in independent variable will give one value either for the regression slope or its reciprocal and will vary the margin of error for that slope resulting in different widths for the confidence intervals.

**Fig. 6:**
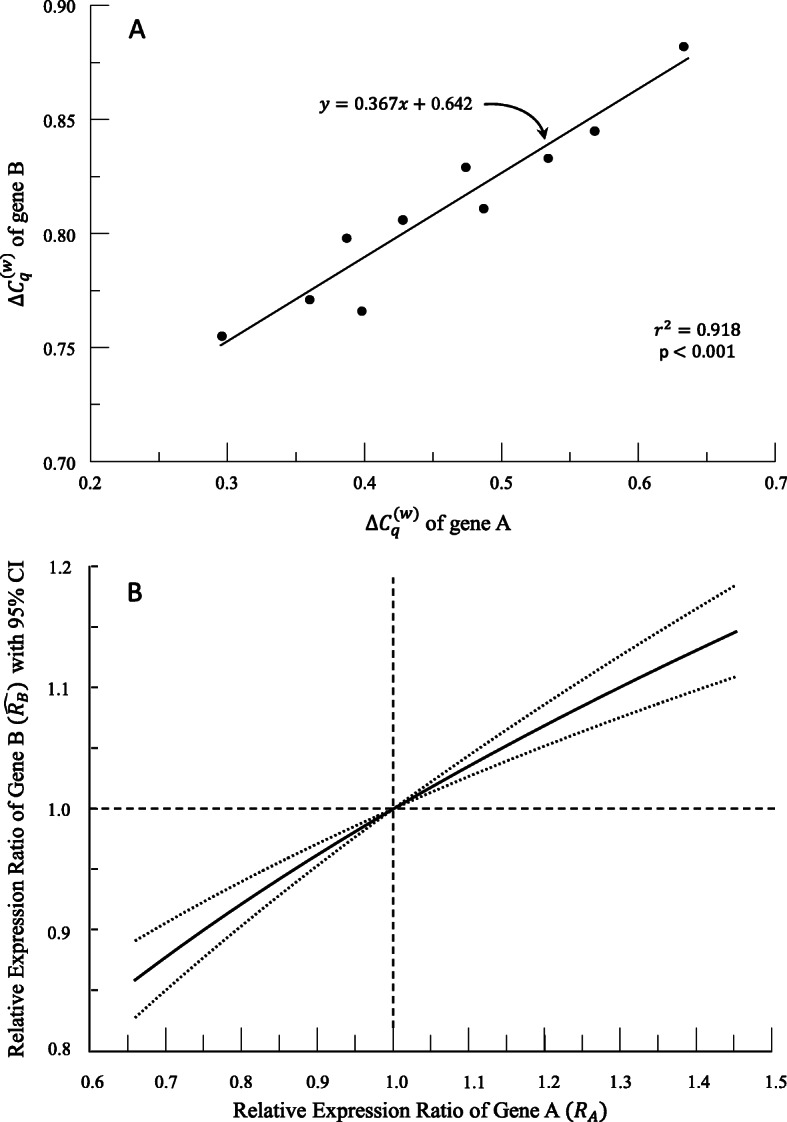
(A) Results of regression between $\Delta  {C}_q^{(w)}$ of gene B and $\Delta  {C}_q^{(w)}$ of gene A. (B) Plot of predicted relative expression ratio of gene B ($\hat{R_B}$ ) with 95% confidence interval (CI) based on relative expression ratio of gene A ($\hat{R_A}$ ) with 95% confidence interval (CI). *x*_0_ value is the mean *x*

Suppose that the independent variable *x* is given by ${\Delta  C}_{q;A}^{(w)}$ describing expression of gene A and the dependent variable *y* is given by ${\Delta  C}_{q;B}^{(w)}$ describing expression of gene B. Suppose that a valid linear regression (Figure 6A, Table 5) has produced the formula

17 $$ \hat{y}=\hat{{\Delta C}_{q;B}^{(w)}}=m\ast {\Delta C}_{q;A}^{(w)}+b= mx+b $$

**Table 5:** Hypothetical data used to generate Figure 6A. Calculation of predicted relative expression, $\hat{R_B}$ , values follows ${R}_A^m$, where *m* = 0.367, and these values are plotted in Figure 6B. *x*_0_ = 0.457 is the mean *x*. The 95% confidence interval for the slope *m* is (0.278, 0.456)

${\Delta C}_{q;A}^{(w)}$	${\Delta C}_{q;B}^{(w)}$	${\Delta \Delta C}_{q;A}^{(w)}$	*R*_*A*_	$\hat{R_B}$	Lower Confidence Intervals	Upper Confidence Intervals
0.633	0.882	0.177	0.666	0.861	0.831	0.893
0.568	0.845	0.112	0.774	0.910	0.889	0.931
0.534	0.833	0.078	0.837	0.937	0.922	0.952
0.474	0.829	0.018	0.961	0.985	0.982	0.989
0.487	0.811	0.031	0.932	0.975	0.968	0.981
0.428	0.806	−0.029	1.068	1.024	1.018	1.030
0.387	0.798	− 0.070	1.174	1.061	1.045	1.076
0.398	0.766	−0.059	1.144	1.051	1.038	1.063
0.360	0.771	−0.097	1.249	1.085	1.064	1.107
0.296	0.755	−0.161	1.447	1.145	1.108	1.184

We fix a baseline level for ${\Delta  C}_{q;A}^{(w)}$, which we label as *x*_0_, and get $\hat{y_0}=m{x}_0+b$ as usual. Given ${\Delta  C}_{q;A}^{(w)}=x-{x}_0$, we then subtract $\hat{y_0}=m{x}_0+b$ from (equation 17) and use notation similar to (equation 12) for gene A and B to produce

18 $$ \hat{{\Delta \Delta C}_{q;B}^{(w)}}=\hat{y}-\hat{y_0}=m\left(x-{x}_0\right)=m\Delta {\Delta C}_{q;A}^{(w)} $$

Applying an exponential function to both sides and applying some algebra reveal

19 $$ \boxed{\hat{R_B}}={10}^{-\Delta \hat{{\Delta C}_{q;B}^{(w)}}}={10}^{-m\Delta {\Delta C}_{q;A}^{(w)}}={\left({10}^{-\Delta {\Delta C}_{q;A}^{(w)}}\right)}^m\boxed{={R}_A^m} $$

showing that the relative expression ratio for B is the m^th^ power of the relative expression ratio for A in this case (Figure 6B, Table 5). From our example, individuals with 10% higher expression of gene A (*R*_*A*_ = 1.1) are predicted to express gene B at a 3.6% higher rate ($\hat{R_B}=1.036$) relative to individuals with average gene A expression.

Yet again we can generate formulas for confidence intervals for each value of $\hat{R_B}$ predicted by a given value of *R*_*A*_. As in all earlier cases, all uncertainty derives directly from the uncertainty in the slope parameter. Suppose that the confidence interval on slope *m* is *(L, U).* Substitute this expression into (equation 19) and simplify to calculate a confidence interval for $\hat{R_B}$ based on a specified value of *R*_*A*_ (Figure 6B, Table 5).

20 $$ \boxed{\mathrm{CI}\ \mathrm{for}\ \hat{R_B}=\left({R}_A^L,{R}_A^U\right)\ \mathrm{or}\ \left({R}_A^U,{R}_A^L\right)} $$

depending upon whether *R*_*A*_ > 1 or 0 < *R*_*A*_ < 1. For our example, the 95% confidence interval around $\hat{R_B}$ is 1.027–1.044, which corresponds to a predicted expression of gene B at 2.7–4.4% higher than that of individuals with average gene A expression.

### A note on the assumption of linearity

There are important assumptions that must be met for regression analysis to be considered appropriate. These assumptions are covered in any general statistics text, and so we omit them here to conserve space. However, one of these assumptions, that of linearity, is worth discussing further. All of the work above assumes that there is a linear relationship between variable *x* and ${\Delta  C}_q^{(w)}$, ${\Delta  C}_q^{(w)}$ and variable *y*, or between ${\Delta  C}_{q;A}^{(w)}$ and ${\Delta  C}_{q;B}^{(w)}$. In these cases, the linear relationship between *y* and *x* resulted in either an exponential relationship between relative expression ratio *R* and *x*, a logarithmic relationship between *R* and *y*, or a power relationship between *R*_*A*_ and *R*_*B*_. Theoretically, the functional relationships between measured variables and measures of gene expression (in our case the efficiency-weighted *C*_*q*_, ${\Delta  C}_q^{(w)}$) could assume any number of shapes depending on the gene of interest, the experimental condition, and even the species [5, 20], leading to other functional relationships between *R* and *x*, *R* and *y*, and *R*_*A*_ and *R*_*B*_. In cases where *x* and *y* are not linearly related, it is common to apply transformations to the data to improve linearity. A properly chosen transformation can allow for the linearity assumption to be met and a linear regression to be performed. However, the mathematical approach to calculating *R* is constrained by the specific transformation that was chosen.

The common base method is amenable for considering many functional types; however, for this paper we focus on only a few cases that we hope will illustrate the general concept. Above, we developed the calculations for the relationship between relative expression ratio *R* and an independent variable *x* that is exponential (*R* = *kb*^*x*^) when ${\Delta  C}_q^{(w)}$ and *x* are linearly related. We also developed a logarithmic formula y = a + b*log*(R)* for linear relationships between a dependent variable *y* and *R* when they are linearly related*.* We finally showed that a power function (${R}_B={R}_A^m$) results when ${\Delta  C}_{q;A}^{(w)}$ and ${\Delta  C}_{q;B}^{(w)}$ are linearly related.

${\Delta  C}_q^{(w)}$ as the Dependent Variable and Log-Transformed *x*.

Earlier we showed how linear relationships between ${\Delta  C}_q^{(w)}$ and another variable resulted in exponential or logarithmic relationships. We now develop the calculations to show that power functions (*R* = *kx*^*a*^), including linear proportions (*R* = *kx*) as a special case when *a* = 1, occur when ${\Delta  C}_q^{(w)}$ and log(*x*) have a linear relationship. Suppose that such a linear relationship exists.

21 $$ {\Delta C}_q^{(w)}=m\log (x)+b $$

**Fig. 7:**
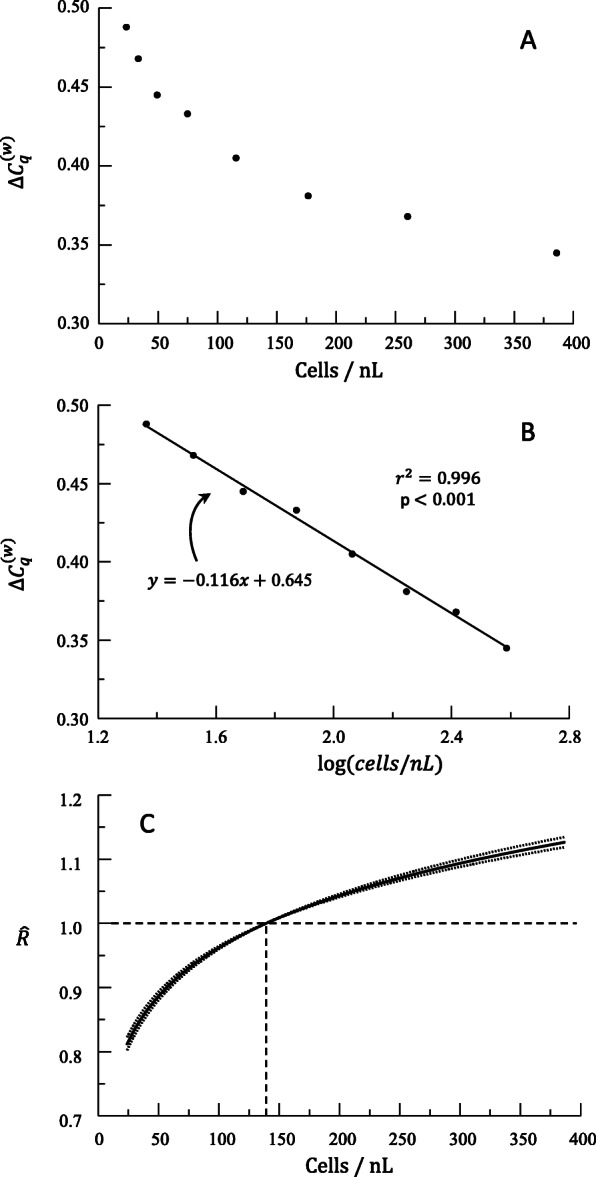
(A) Plot of log rhythmic relationship between cells / nL and $\Delta  {C}_q^{(w)}$. (B) Results of regression analysis with *log*(*cells*/*nL*) and $\Delta  {C}_q^{(w)}$. (C) Plot of predicted relative expression ratio ($\hat{R}$) as a function of *log*(*cells*/*nL*) with 95% confidence interval. *x*_0_ value (vertical dotted line) is the mean *x*

In other words, suppose that the relationship between *x* and *y* is logarithmic (Figure 7A). Such plots are linearized by log-transformation of *x* (Figure 7B, Table 6). For example, suppose that expression of a particular bacterial gene is predicted by the density of the bacteria in culture. The function relating ${\Delta C}_q^{(w)}$ to density of cells shows that ${\Delta C}_q^{(w)}$ responds more to a change in density when the bacterial count is low than when the bacterial count is high.

**Table 6:** Hypothetical data used to generate Figure 7A, B. Calculation of predicted relative expression, $\hat{R}$, values follows ${\left(\frac{x_0}{x}\right)}^m$, where *m* =  − 0.116, and these values are plotted in Figure 7C. *x*_0_ = 139.8 is the mean *x*. The 95% confidence interval for the slope *m* is (−0.123, −0.109)

Cells / nL	*log*(*cells*/*nL*)	${\Delta C}_q^{(w)}$	$\hat{R}$	Lower Confidence Interval	Upper Confidence Interval
385.61	2.586	0.345	1.125	1.117	1.133
260.29	2.415	0.368	1.075	1.070	1.079
176.41	2.247	0.381	1.027	1.026	1.029
115.56	2.063	0.405	0.978	0.977	0.979
74.72	1.873	0.433	0.930	0.926	0.934
49.29	1.693	0.445	0.886	0.880	0.893
33.37	1.523	0.468	0.847	0.838	0.855
23.12	1.364	0.488	0.812	0.801	0.822

Suppose then that log(*x*) (log (number of cells / nL)) and *y* (${\Delta C}_q^{(w)}$) fit a linear relationship with the line of best fit

22 $$ \hat{{\Delta C}_q^{(w)}}=\hat{y}=m\log (x)+b $$

We again choose a fixed baseline value *x*_0_ for the variable *x* and subtract equations using inputs *x* and *x*_0_ as we did with (equation 5) and (equation 6) yielding

23 $$ \hat{\Delta {\Delta C}_q^{(w)}}=\hat{y}-\hat{y_0}=m\left(\log (x)-\log \left({x}_0\right)\right) $$

After applying the exponential transformation, we have

24 $$ \hat{R}={10}^{-\hat{\Delta {\Delta C}_q^{(w)}}}={10}^{-m\left(\log (x)-\log \left({x}_0\right)\right)}={10}^{m\left(\log \left({x}_0\right)-\log (x)\right)} $$

Using algebraic properties of the logarithm, we produce

25 $$ \hat{R}={10}^{m\left(\log \left({x}_0\right)-\log (x)\right)}={10}^{m\log \left(\frac{x_0}{x}\right)}={10}^{\log \left[{\left(\frac{x_0}{x}\right)}^m\right]}={\left(\frac{x_0}{x}\right)}^m $$

In conclusion, when efficiency-weighted ${\Delta C}_q^{(w)}$ values have a logarithmic relationship to *x*, then we obtain a power function relationship between relative expression ratio *R* and *x* (Figure 7C, Table 6).

26 $$ \boxed{\hat{R}={\left(\frac{x_0}{x}\right)}^m} $$

Again, notice that inputting a concentration of hormone α_1_ *x* = *x*_0_ will result in a predicted relative expression ratio of 1 as we would expect.

**Fig. 8:**
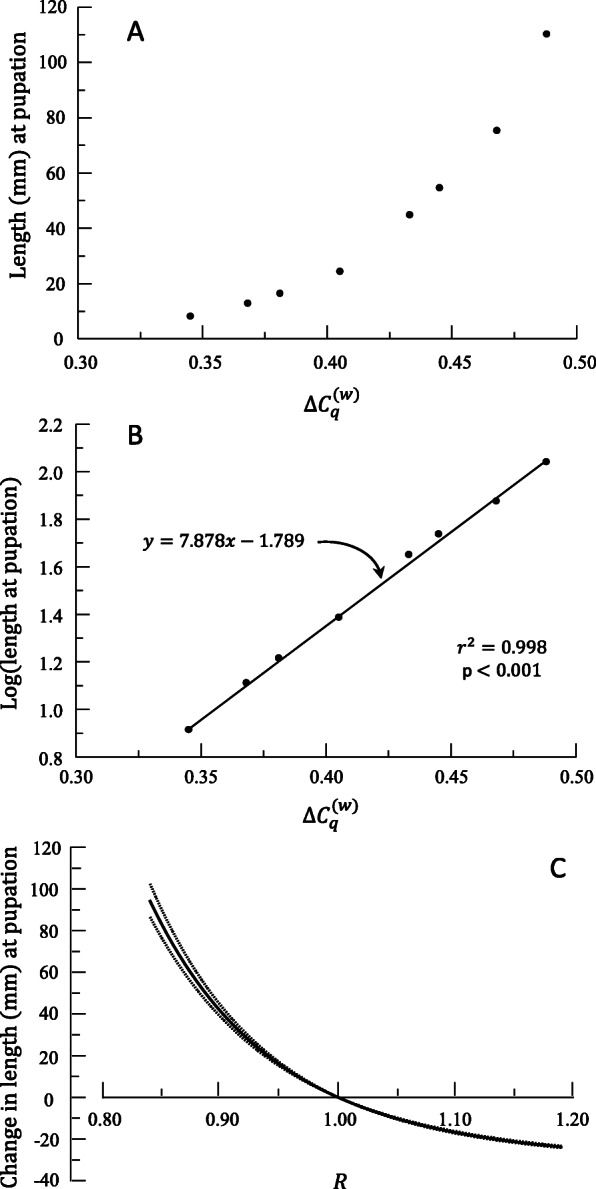
(A) Plot of exponential relationship between $\Delta  {C}_q^{(w)}$ and *y*. (B) Results of regression analysis with $\Delta  {C}_q^{(w)}$ and *log*(*y*). (C) Plot of predicted *y*, $\hat{y}$, as a function of relative expression ratio with 95% confidence interval. *x*_0_ value is the mean *x*

In the case where log(*x*) and ${\Delta C}_q^{(w)}$ are linearly related, the process for calculating a confidence interval only needs slight alterations compared to our first case. By tracking (equations 9, 23—27), we see that appending log() around each *x* or *x*_0_ will result in the correct formula. Therefore, we adjust (equation 10) and apply some algebraic properties of logarithms (as in (equation 26)) to obtain:

27 $$ \boxed{\mathrm{CI}\ \mathrm{for}\ \hat{R}=\left({\left(\frac{x_0}{x}\right)}^L,{\left(\frac{x_0}{x}\right)}^U\right)\ \mathrm{or}\ \left({\left(\frac{x_0}{x}\right)}^U,{\left(\frac{x_0}{x}\right)}^L\right)} $$

depending upon whether the ratio $\frac{x_0}{x}$ is greater than 1 or less than 1 for each value of *x*, which in turn is equivalent to whether (*x* − *x*_0_) is positive or negative (Figure 8C). From our example above (Table 6), a concentration of cells of 70 cells / nL would be predicted to have a 7.7% lower expression ($\hat{R}=0.923\Big)$ than cells at the average concentration of 140 cells / nL with a 95% CI of a decrease in expression of 7.3–8.2%.

${\Delta  C}_q^{(w)}$ as the Independent Variable and Log-Transformed *y*

Where the relationship between *x* and *y* is log-linear (Figure 8A, Table 7), it may be necessary to log transform the dependent *y* values to establish a linear relationship with ${\Delta  C}_q^{(w)}$ as the independent variable (Figure 8B). For example, in a species of insect, a particular gene is implicated in determining the size at pupation. Slight changes in gene expression at high expression levels have minimal effects on the size at pupation. However, at lower levels of expression, small changes in expression have disproportionate effects.

**Table 7:** Hypothetical data used to generate Figure 8A, B. Calculation of predicted *y*, $\hat{y}$, values follows $\hat{y_0}\kern0.50em {R}^{-m}$, where *m* = 7.878 and $\hat{y_0}=31.094$, and these values are plotted in Figure 8C. *x*_0_ = 0.417 is the mean *x*. The 95% confidence interval for the slope *m* is (7.516, 8.241)

${\Delta C}_q^{(w)}$	Larval length at pupation	Log(larval length at pupation)	*R*	$\hat{y}$	Lower Confidence Interval	Upper Confidence Interval
0.345	8.23	0.915	1.180	8.423	7.931	8.944
0.368	12.96	1.112	1.119	12.784	12.271	13.317
0.381	16.49	1.217	1.086	16.183	15.704	16.676
0.405	24.44	1.388	1.028	25.012	24.762	25.263
0.433	44.89	1.652	0.964	41.565	41.014	42.124
0.445	54.76	1.738	0.938	51.673	50.481	52.896
0.468	75.45	1.878	0.889	78.425	75.161	81.840
0.488	110.39	2.043	0.849	112.724	106.246	119.616

Suppose that the assumptions for a valid linear regression have been met with a line of best fit

28 $$ \log \left(\hat{y}\right)=m\ast {\Delta  C}_q^{(w)}+b= mx+b $$

Again, one should judiciously choose a baseline value for gene expression ${\Delta  C}_q^{(w)}$, which we label as *x*_0_. We again set

29 $$ {\Delta \Delta  C}_q^{(w)}={\Delta  C}_q^{(w)}-{x}_0 $$

and have $\log \left(\hat{y_0}\right)=m{x}_0+b$. Thus,

30 $$ \boxed{\hat{y_0}={10}^{\left(m\ast {x}_0+b\right)}} $$

Subtracting the equation for $\log \left(\hat{y_0}\right)$ from (equation 29) yields the formula

31 $$ \log \left(\hat{y}\right)-\log \left(\hat{y_0}\right)=m\left(x-{x}_0\right)=m{\Delta \Delta C}_q^{(w)} $$

We apply some logarithmic properties to obtain the following:

32 $$ \log \left(\frac{\hat{y}}{\hat{y_0}}\right)=\log \left(\hat{y}\right)-\log \left(\hat{y_0}\right)=m{\Delta \Delta C}_q^{(w)} $$

Next, apply the exponential function.

33 $$ \frac{\hat{y}}{\hat{y_0}}={10}^{m{\Delta \Delta C}_q^{(w)}}={\left({10}^{-\Delta \Delta {C}_q^{(w)}}\right)}^{-m}={R}^{-m} $$

Finally, solve for $\hat{y}$ to obtain the power function (Figure 8C, Table 7):

34 $$ \boxed{\hat{y}=\hat{y_0}{R}^{-m}} $$

This equation tells us that for a given *R*, or relative expression ratio between two values, we expect a specific change in response variable *y* (Figure 8C, Table 7). We can generate formulas for confidence intervals to place around predicted values of the dependent variable given values of *R.* Suppose that the confidence interval on the slope parameter *m* is *(L, U).* Substitute this expression into (equation 35) and simplify to calculate a confidence interval for $\hat{y}$ based on a specified value of *R*.

35 $$ \boxed{\mathrm{CI}\ \mathrm{for}\ \hat{y}=\left(\hat{y_0}{R}^{-L},\hat{y_0}{R}^{-U}\right)\ \mathrm{or}\ \left(\hat{y_0}{R}^{-U},\hat{y_0}{R}^{-\mathrm{L}}\right)} $$

depending on whichever interval is in the correct order. Given our example, a 10% higher level of expression (*R* = 1.1) predicts a decrease in length of larvae at pupation from 16.4 mm to 14.7 mm. The 95% CI for the length of the larva at pupation is 14.2–15.2 mm when expression is 10% higher than individuals with average expression. Note that these results are only applicable with the currently chosen *x*_0_.

### Other cases

While we treated cases above where the non-gene variable needed to be log-transformed first to establish a linear relationship, we have not discussed cases where ${\Delta  C}_q^{(w)}$ needs such a log-transformation. Although we omit the derivations to conserve space, placing ${\Delta  C}_q^{(w)}$ inside of a logarithmic function, setting up a ${\Delta \Delta  C}_q^{(w)}$ formula, and then manipulating to convert ${\Delta \Delta  C}_q^{(w)}$ into relative expression ratio *R* will yield functional formulas that are “doubly exponential” or “doubly logarithmic.” While such formulas are not impossible, they do not appear to be common in nature. Another way to consider this situation is that since $R={10}^{-\Delta {\Delta  C}_q^{(w)}}$ with ${\Delta \Delta  C}_q^{(w)}$ in the exponent of *R*, we can view ${\Delta \Delta  C}_q^{(w)}$ as something that is already derived through a log-transformation applied to *R*. Thus, applying a logarithm to ${\Delta  C}_q^{(w)}$ would be like applying two layers of log transformations to *R*, which does not seem likely to be necessary.

**Fig. 9:**
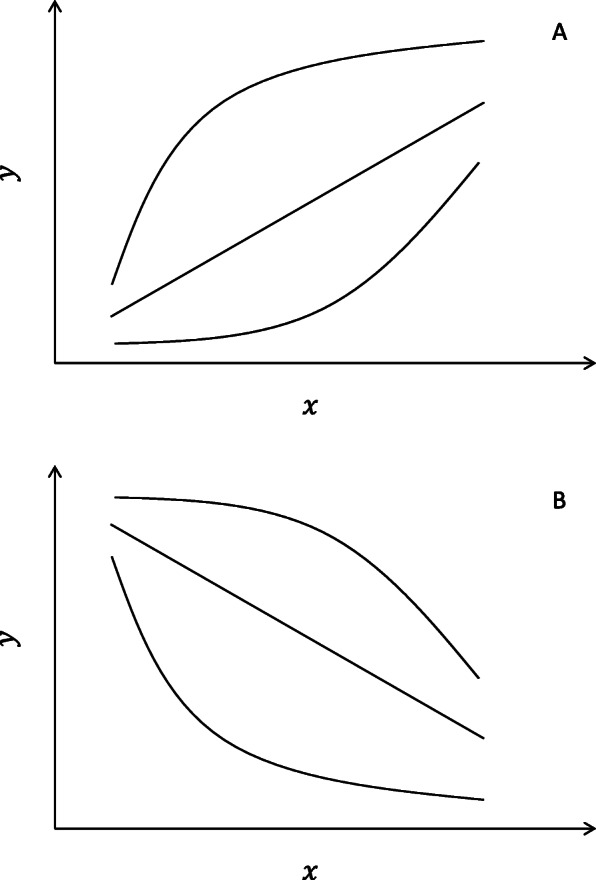
(A, B) Approximate curve shapes covered in this paper by the common base method

On the other hand, one should not view an omission of any particular functional form in this work to represent a dismissal of that form as impossible. Nevertheless, our treatment of linear, exponential, logarithmic, and power forms covers the most common functional relationships curve shapes for two variables (Figure 9).

### Analysis of covariance

The common base method [18] may be used to perform paired and unpaired 2-sample *t*-tests and calculate 2-sample *t*-intervals as well as analysis of variance (ANOVA). These approaches can fail, however, when the quantities being compared between the groups are also affected by an uncontrolled quantitative covariate. In that case, analysis of covariance (ANCOVA) is a powerful analysis tool that combines ANOVA and linear regression techniques. In a simple, one-way ANCOVA, there will be three variables of interest: the factor or treatment effect (an independent categorical variable consisting of at least two groups), the response (a dependent quantitative variable), and a covariate (an independent quantitative variable).

For example, suppose that we have determined that ${\Delta  C}_q^{(w)}$ of a gene *RT* in larvae is affected by temperature. We might have a suspicion that *RT* expression is also affected by the larvae’s diet. We could perform an experiment at a single temperature where larvae are given an experimental and control diet. This would be a traditional use of qPCR and can be analyzed with the common base method as a 2-sample *t*-test. However, since we already know that temperature affects *RT*, we would be left wondering if the diet change was effective in altering *RT* expression across temperatures or if temperature and diet interact in some fashion. We could design an experiment that looks at both temperature and diet at the same time. Instead of designing an experiment with several larvae (replicates) in each combination of temperature and diet (two-factor ANOVA), we will instead grow larvae in three treatments: two experimental diets and one control diet across a range of temperatures (the covariate) in order to analyze the effect on expression of *RT* (the response).

Since we know from previous research that temperature and ${\Delta  C}_q^{(w)}$ of *RT* are related linearly, we really are not interested in performing another experiment to test this hypothesis. Instead we are interested in the effect of diet on ${\Delta  C}_q^{(w)}$ of *RT,* and we can determine if this effect is similar across temperatures or whether diet and temperature interact to alter ${\Delta  C}_q^{(w)}$ of *RT*. An ANCOVA is the obvious choice to test this hypothesis. Note that in our example above, temperature is manipulated by the researcher. However, covariates may also be unmanipulated variables that vary among individuals that are known to affect *y*.

### The basic process for ANCOVA

(1) Perform separate linear regressions on the response as a function of the covariate for each of the treatment groups, and determine that at least one of those lines has a slope statistically different from zero. (If all slopes are zero, then the covariate may be ignored, and ordinary ANOVA used instead.)

(2) Verify homogeneity of slopes for the lines. Although it is unlikely that the regression step produced lines with identical slopes, it is possible that the data fit a model with an enforced common slope. Testing homogeneity of slopes relies on testing the significance of the interaction term between the treatment and covariate, diet*temperature in our example. Depending upon your choice of software, you will probably run some form of fit for a general linear model (possibly within an ANOVA menu) that accepts a response, treatment, and covariate. Often in an option for “model,” you can enter the interaction term. The resulting output should include a *p*-value for the interaction. The p-value for this interaction tests a null hypothesis that the slopes are the same. If the p-value is greater than 0.05, then you fail to reject the null hypothesis and may assume the slopes are homogeneous. If the p-value is smaller than 0.05, then the interaction between the treatment and covariate is significant, and so the slopes of the lines are likely different. In this case, ANCOVA is not appropriate.

(3) Where slopes are homogeneous, rerun the general linear model routine but without the interaction term in order to recalculate the regression lines with a new enforced common slope. Most software packages should also offer options for “contrasts” or “comparisons” that will generate confidence intervals for pairwise comparisons between treatments. We will avoid dictating which of the many types of contrasts (Fisher, Tukey, Sidak, or Bonferonni) is preferable.

### Relative expression ratios and confidence intervals from ANCOVA

Suppose that all three steps above have gone correctly and that for the three treatments we now have regression lines that share an enforced common slope. Notice that the slope, *m*, is the same for each equation.

36 $$ \hat{y}= mx+{b}_1,\hat{y}= mx+{b}_2,\mathrm{and}\ \hat{y}= mx+{b}_3 $$

Then the differences in the lines are measured by *b*_2_ − *b*_1_, *b*_3_ − *b*_1_, and *b*_3_ − *b*_2_ respectively.

**Fig. 10:**
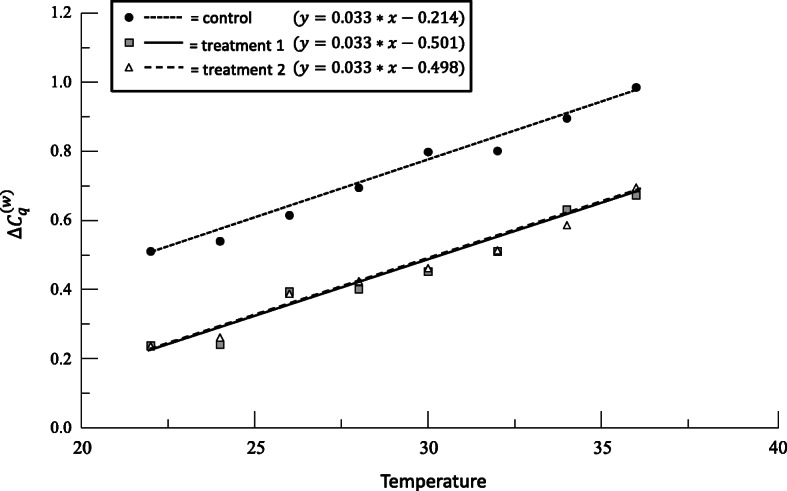
ANCOVA plot using data from Table 8. Plotted regression lines use the common slope of 0.033

In our example, *x* stands for temperature while *y* stands for the ${\Delta  C}_q^{(w)}$ of *RT*. We use the subscripts *c* to denote control diet and *t*1 and *t*2 to denote treatment diets. Since the lines have the same slope, they are all parallel, and each pair has a constant vertical difference given by the difference between intercept values: *b*_*t*1_ − *b*_*c*_, *b*_*t*2_ − *b*_*c*_, and *b*_*t*2_ − *b*_*t*1_. As that difference is a measurement on the *y*-scale, it represents a predicted $\hat{\Delta {\Delta C}_q^{(w)}}$ measurement (Figure 10). For example, *b*_*t*1_ − *b*_*c*_ and its confidence interval predict the effect on ${\Delta  C}_q^{(w)}$ between treatment1 and the control at any given value *x* of the covariate. In our example, we are calculating the effect that the two different diets have on expression of the gene *RT* while controlling for temperature.

We may now calculate a predicted relative expression ratio $\hat{R}$ showing the difference in any pair of factors (e.g., treatment1 effect relative to the control on the gene) at any given covariate value.

37 $$ \boxed{\hat{R}}={10}^{-\hat{\Delta {\Delta C}_q^{(w)}}}\boxed{={10}^{-\left({b}_i-{b}_j\right)}} $$

Similar to our regression analysis, we may also calculate a confidence interval for this predicted relative expression ratio using (equation 38) and the confidence interval *(L,U)* calculated for the difference *b*_*i*_ − *b*_*j*_ between any two factors.

38 $$ \boxed{\mathrm{CI}\ \mathrm{for}\ \hat{R}=\left(\ {10}^{-U},{10}^{-L}\ \right)} $$

where the order of *L* and *U* has switched because of the negative multiplier in the exponential function.

**Fig. 11:**
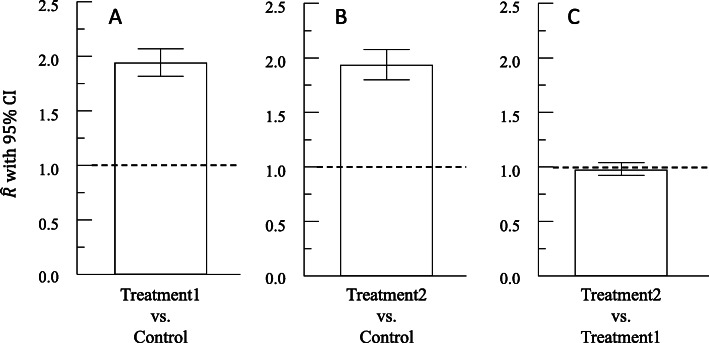
Relative expression values with 95% confidence intervals for each post-hoc comparison (A, B, C)

For our example data (Table 8), a check of the homogeneity of slopes assumption shows that we can treat our lines as parallel (*p* = 0.613). Rerunning the analysis without the interaction term shows that both temperature and diet affect ${\Delta  C}_q^{(w)}$. Post-hoc analysis shows that the treatment diets were both significantly different from the control (*p* < 0.001), but the two treatment diets were not different from each other (*p* = 0.829). Larvae exposed to the treatment1 diet expressed *RT* at a level 194% higher than in the control (95% CI = 181—207%; Figure 11). Larvae exposed to the treatment2 diet expressed *RT* at a level 192% higher than in the control (95% CI = 181—207%; Figure 11). With no difference in *RT* expression between the two treatments the 95% CI for relative expression comparing treatment2 to treatment1 ($\hat{R}$ = 0.993) overlaps 1 with the 95% CI = 0.930—1.061 (Figure 11).

**Table 8:** Hypothetical data used to generate Figures 10, 11. Calculation of relative expression follows ${10}^{-\left({b}_1-{b}_2\right)}$, where *b* represents the *y* intercept, and the subscripts *c*, *t*1, and *t*2 represent control, treatment1, and treatment2 respectively

Treatment	Temperature (°C)	${\Delta C}_q^{(w)}$			
Control	22	0.511		*b*_*t*1_ − *b*_*c*_	−0.2873
Control	24	0.540		Lower CI	−0.3159
Control	26	0.615		Upper CI	−0.2586
Control	28	0.694		$\hat{R}$	1.938
Control	30	0.798		Upper $\hat{R}$	1.814
Control	32	0.801		Lower $\hat{R}$	2.070
Control	34	0.895			
Control	36	0.985			
Treatment1	22	0.238		*b*_*t*2_ − *b*_*c*_	−0.2843
Treatment1	24	0.241		Lower CI	−0.3129
Treatment1	26	0.394		Upper CI	−0.2556
Treatment1	28	0.401		$\hat{R}$	1.924
Treatment1	30	0.452		Upper $\hat{R}$	1.801
Treatment1	32	0.511		Lower $\hat{R}$	2.055
Treatment1	34	0.631			
Treatment1	36	0.673			
Treatment2	22	0.236		*b*_*t*2_ − *b*_*t*1_	0.0030
Treatment2	24	0.261		Lower CI	−0.0257
Treatment2	26	0.388		Upper CI	0.0317
Treatment2	28	0.424		$\hat{R}$	0.993
Treatment2	30	0.462		Upper $\hat{R}$	0.930
Treatment2	32	0.513		Lower $\hat{R}$	1.061
Treatment2	34	0.586			
Treatment2	36	0.695

One of the key assumptions of the ANCOVA process is that the slopes of the regression lines can be statistically treated as equal, even if they are not calculated to be exactly equal during individual regression analysis. The analysis generates a common slope for each trend line, and the differences between the intercepts derive from these rather than the original slope estimates. In our example above, the common slope is estimated to be 0.033. If this homogeneity assumption does not hold, then the ANCOVA cannot proceed as there is evidence that the difference between the lines is not constant with respect to the covariate.

## Discussion

As you work through this approach, there are important things to consider.

1. It is preferable that the ${\Delta  C}_q^{(w)}$ values should be derived from efficiency (*E*) and *C*_*q*_ values from a single qPCR plate. Alternatively, each ${\Delta  C}_q^{(w)}$ value could be derived from a separate qPCR plate. The issue, though, is unexplained variation. Where ${\Delta  C}_q^{(w)}$ values derive from different plates, differences between these values may be attributable to differences among individuals, qPCR plates, wells on the plate, and the independent variable. Where ${\Delta  C}_q^{(w)}$ values derive from a single qPCR plate, variation is attributable to difference among individuals, wells on the plate, and the independent variable. If several ${\Delta  C}_q^{(w)}$ values are derived from a single qPCR plate, while several other values are derived from a second plate, then we cannot partition variation attributable to plate. The result, then, statistically is to increase the unexplained variation (reduce *r*^2^), which in turn increases our confidence intervals around our *y* estimates. Determining significance is more difficult where such an effect exists.

2. For production of the relative expression plots, only use *x* values within the range of *x* values used in the study or experiment.

3. Production of the linear equation through regression analysis allows us to determine *y* values given *x* values. Interpretation of this relationship depends upon the experimental design. Where *x* values are measured from randomly chosen individuals (unmanipulated), the relationship is predictive but not necessarily causal. Care should be exercised in such interpretations. Where *x* values are manipulated as part of an experiment, it may be appropriate to apply such causality.

4. Presentation of relative expression values should be accompanied by confidence intervals [18]. It is not enough to report the relative expression value since, depending on the tightness of the relationship, confidence can vary greatly.

5. Relative expression plots are based on an inverse axis—high ${\Delta  C}_q^{(w)}$ values represent lower expression than low ${\Delta  C}_q^{(w)}$ values. As such, all *R* plots should be interpreted with care.

6. It is important to check all of the assumptions for performing a linear regression. For publication, it is important for readers to see the regression relating ${\Delta  C}_q^{(w)}$ values to another variable. This allows readers to assess the linearity assumption. The *R* plot containing confidence intervals should also be presented for linear regression analyses. For ANCOVA results, the plot of ${\Delta  C}_q^{(w)}$ values by treatment against the covariate is valuable. Part of the calculation of ${\Delta  \Delta  C}_q^{(w)}$ is to use *b*_1_ − *b*_2_. The difference between the *y*-intercepts is actually equal to the difference between the two regression lines at the average covariate value.

7. The experimental design and statistical approach should be addressed explicitly in the methods section. How are the ${\Delta  C}_q^{(w)}$ values analyzed? How are the ${\Delta  C}_q^{(w)}$ values manipulated to yield ${\Delta  \Delta  C}_q^{(w)}$ values and ultimately yield relative expression values with associated confidence intervals? All too often such explanations are neglected, making it very difficult to evaluate the quality of the research.

## Conclusion

Traditional qPCR analysis is not able to address statistical models other than the paired *t*-test. The common base method is amenable for use with any of the statistical models from the general linear model. Here we have shown how the common base method may be applied to determine relationships between ${\Delta  C}_q^{(w)}$ values and an independent variable, a dependent variable, or another gene’s ${\Delta  C}_q^{(w)}$ values. We have developed the concept of how to plot relative expression ratios *R* compared to an untransformed or log-transformed dependent or independent variable or to another relative expression ratio. In this manner, we can predict either how relative expression will change given a change in a measured variable, how a measured variable will change given an experimental change in expression, or how expression will change given a change in expression of a second gene.

## Methods

### Regression

In a simple linear regression analysis, we are attempting to determine if a linear relationship exists between two variables and, if so, describe the relationship. A linear regression analysis will return a linear equation *y* = *mx* + *b* connecting the two variables *x* and *y*. The analysis will at a minimum yield a coefficient of determination *r*^2^ and a *p*-value associated with the slope test. The *r*^2^ value is a number between 0 and 1 that indicates the amount of variation in *y* that can be explained by variation in *x.* The closer *r*^2^ is to 1, the better the linear relationship or fit between the two variables. The p-value is used to test whether or not the slope *m* is significantly different from zero.

In the results section we describe cases of linear regression where one of the variables is the efficiency-weighted ${C}_q,{\Delta  C}_q^{(w)}$. The ultimate goal will then be to show how such a regression line can be transformed into a nonlinear formula where one of the variables is a relative expression ratio *R*. To our best knowledge, conceptualization of relative expression ratios in this manner is novel.

## Data Availability

All data used are available in the manuscript.

## Abbreviations

ANCOVA: Analysis of covariance
ANOVA: Analysis of variance
GOI: Gene of interest
qPCR: Quantitative polymerase chain reaction
*R*: Relative expression value
REF: Reference gene

## Footnotes

1 The choice of baseline *x*_0_ will be discussed in a later section.

2 In linear regression, it is standard to have both confidence intervals and prediction intervals. We have chosen to use confidence intervals, but everything that we have developed can be used to calculate prediction intervals.

3 As the cases of ${\Delta  C}_q^{(w)}$ as dependent variable and ${\Delta  C}_q^{(w)}$ as independent variable are inverses, they each present essentially the same information but in two different manners. The nature of the experiment should help guide which approach is preferred. We advocate using ${\Delta  C}_q^{(w)}$ as the independent variable only in situations where ${\Delta  C}_q^{(w)}$ is a manipulated variable, i.e., the experimental design manipulated the level of some gene’s expression. Otherwise, we suggest relegating ${\Delta  C}_q^{(w)}$ to the dependent variable. When ${\Delta  C}_q^{(w)}$ is a dependent variable, you will be able to calculate a predicted relative expression ratio from a given input value *x*. When ${\Delta  C}_q^{(w)}$ is the independent variable, you will only be able to calculate a predicted *change* in variable *y* compared to a predicted baseline given an input relative expression ratio, instead of predicting an *absolute* calculation for *y*. The former situation is slightly easier to plot and describe.
